# Effect of Supplemental Trace Mineral Source on Haircoat and Hair Loss in Adult Cats

**DOI:** 10.3390/ani15182662

**Published:** 2025-09-11

**Authors:** Laura A. Amundson, Allison A. Millican, Alyssa S. Cornelison, Michael L. McGilliard, Taryn Matti

**Affiliations:** 1 Zinpro Corporation, 7500 Flying Cloud Drive, Eden Prairie, MN 55344, USA; amillican@zinpro.com (A.A.M.); acornelison@zinpro.com (A.S.C.); 2 Virginia Tech, School of Animal Sciences, Blacksburg, VA 24061, USA; mike.mcgilliard@gmail.com; 3 Summit Ridge Farms, 4526 State Route 2073, Susquehanna, PA 18847, USA

**Keywords:** complexed organic trace minerals, domestic cats, skin, shed hair, dander

## Abstract

Trace minerals (TMs) are pivotal for skin health and haircoat quality. The objective of this study was to evaluate effects of organic TMs, in the form of lysine and glutamic acid-complexed TMs (Zn, Mn, Cu, and Fe), on feline hair growth and haircoat characteristics. Cats (mean 8.4 yr) were fed a Control diet (inorganic TM, sulfates) for 15 days before being assigned to treatment diets for 90 days. Treatments were Control (n = 20) or lysine and glutamic acid-complexed TM (Zinpro Corp. Eden Prairie, MN, USA) sources (TMC-LG; n = 20), formulated at iso-levels (100 ppm Zn, 15 ppm Mn, 12 ppm Cu, 80 ppm Fe). At the beginning of the test period (day 0), an area was shaved to evaluate hair growth on days 45 and 90, in addition to haircoat characteristics and hair loss. At day 45 (diet × day, *p* = 0.02), cats fed TMC-LG tended to have 0.023 g less shed hair compared to Control cats (*p* = 0.08). Similarly, dander scores differed at day 45 (diet × day, *p* = 0.02), with TMC-LG (0.66) cats having more favorable scores compared to Control (1.05; *p* = 0.03). There were no significant differences between treatments at day 90. Cats fed TMC-LG had a transient improvement in hair loss and dander scores at day 45 compared to the Control over the 90-day trial.

## Simple Summary

Trace minerals (TMs) are essential for all animals and are required to maintain healthy skin and coats in companion animals. Supplemental sources of TM vary in their bioavailability and efficacy based on their chemical form. A randomized, controlled 90-day feeding trial was conducted to evaluate the effect of sulfate (inorganic) versus lysine and glutamic acid-complexed TM (organic) sources of Zn, Mn, Cu, and Fe on hair growth and loss, as well as haircoat characteristics, in 40 healthy adult shorthair cats. The results of the study revealed that cats that received the lysine and glutamic acid-complexed TM diet had decreased hair loss (shedding) and improved haircoat scores (less dander) on day 45 but no difference at day 90. Therefore, cats fed lysine and glutamic acid-complexed organic TMs had a transient improvement in skin health and less hair loss. The results reported support the use of TMs from lysine and glutamic acid-complexed organic TMs as a nutritional strategy to maintain healthy skin and coats in cats.

## 1. Introduction

Trace minerals (TMs) are added to complete diets in small quantities and as such are often overlooked. However, they are essential to many structural, physiological, and biochemical functions [1,2,3,4,5]. However, little is known about the role TMs play in the overall wellbeing of the growing companion animal population, especially in cats. While TMs are essential nutrients needed for all living animals, reports have shown that some commercial pet foods contain inadequate or excessive amounts [6,7,8,9], largely due to the unknown TM bioavailability in natural ingredients [8]. Trace mineral bioavailability and metabolism is complex and should be considered when addressing total trace mineral levels in complete diets, where both excesses or inadequacies could be provided by included ingredients [8].

Commercial cat foods are generally supplemented with inorganic TMs; however, it is well known that inorganic and organic TMs are absorbed and utilized differently in the body [10]. Bioavailability, absorption, and utilization are affected by the elemental form and source of the mineral. Organic TMs are classified by the Association of American Feed Control Officials (AAFCO) based on the chelating agent bound to the TM, including metal amino acid complexes, amino acid chelates, metal proteinates, and polysaccharide metal complexes [11]. These classifications can be further defined as trace metals that are chelated to a carbon-containing molecule (amino acids, hydrolyzed proteins, or other carbon molecules). It is important to note that while organic TMs are defined under one single category, properties such as pH, stability, and bioavailability are dependent on the chelating agent and the methods by which the mineral is produced [12]. It is therefore of interest to understand the efficacy of specific organic TMs as they pertain to feline nutrition.

Zinc and Cu provide antioxidant functions and help stabilize proteins, making them less prone to oxidation [13]. Zinc is essential for epithelial tissue development and maintenance, tight junction protein synthesis and function, and to the catalytic, structural, and regulatory functions needed for tissue keratinization [14,15]. Collagen production is pivotal in maintaining skin structure and elasticity, which is dependent on Cu and Mn [16]. The skin is key to maintaining a protective barrier for the animal from the outside environment. Zinc is required for overall growth and is present in every tissue in the body, making it a type II nutrient, and therefore must be consumed daily; it supports skin and mucosal membrane integrity and is thus indispensable for innate and acquired immune function [17]. Additionally, Cu and Mn influence the pro-inflammatory and anti-inflammatory response of macrophages, which modulate the dynamic process of wound healing and biomedical tissue regeneration [18].

Furthermore, phenotypic qualities of skin and haircoat pigmentation are highly regulated through TM supplementation [19]. Zinc has been found to be readily absorbed in dogs when fed as an amino acid chelate versus an oxide, and it accumulates more readily in the coat [10,20]. Another study suggested potential effects on coat health with tendencies for increased glossiness when dogs received an organic source of Zn [21]. Finally, previous work in senior dogs fed amino acid-complexed Zn, Mn, Cu, and Fe significantly improved hair growth and reduced shedding compared to dogs that received sulfates [22]. Thus, the objective of this study was to evaluate the effects of organic TM, in the form of lysine and glutamic acid complexes, on feline skin and haircoat health parameters in adult domestic shorthair cats compared to an inorganic source.

## 2. Materials and Methods

All animal procedures in this study were approved by the Institutional Animal Care and Use Committee at Summit Ridge Farms (Susquehanna, Bala Cynwyd, PA, USA; IACUC—ZPMEFFF00121), in compliance with the Animal Welfare Act [23]. All technicians involved with animal handling, data collection, and data analysis were blinded to treatments.

### 2.1. Animals and Housing

A total of 40 domestic shorthair cats were randomly assigned to one of two groups based on haircoat color, age (5 to 12 years), sex (18 neutered males and 22 spayed females), and body weight (BW; Table 1). All cats were individually identified by a unique ear tattoo and cage card. Inclusion criteria included apparently healthy, spayed/neutered, non-pregnant, and non-lactating adult cats with sufficient hair growth (1 inch or greater) that were cooperative with the proposed study activities. Cats were individually housed in cages that were 63.5 cm W × 119.38 cm L × 121.92 cm H (0.76 sq. meters of floor space) and contained two perches. The cattery was maintained within the target temperature range (50–80 °F) and lighting was on a 12 h light and 12 h dark cycle. Cages, food bowls, water bowls, and litter boxes were cleaned daily and sanitized in accordance with the Animal Welfare Act [23]. All cats were deemed healthy prior to and during the study period by the attending veterinarian at the cattery.

### 2.2. Dietary Treatments

**Figure 1:**
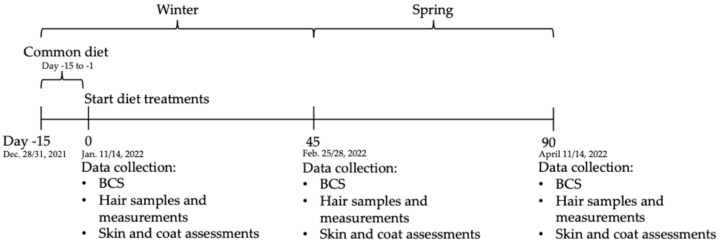
Cat study timeline.

All diets were formulated to meet nutrient requirements for maintenance of adult cats based on the AAFCO recommendations (AAFCO, 2021). All cats received the standard colony diet for the first 14 days of the study period (Purina Cat Chow, Nestlè Purina Petcare USA, St. Louis, MO, USA; −15 to 0 day). At day 0, groups were randomly assigned to either the Control diet containing sulfate minerals (Control, n = 20) or the treatment diet containing lysine and glutamic acid-complexed trace minerals (TMC-LG, n = 20; Zinpro^®^ ProPath^®^, Zinpro Corporation, Eden Prairie, MN, USA) which were fed for 90 days with a staggered start (Table 2; Figure 1). The only variable among treatment diets was the trace mineral premix, with respect to Zn, Mn, Cu, and Fe source. Diets were mixed and extruded according to industry-standard manufacturing practices. The extruded kibble diets were manufactured to meet manufacturer (Mid-South Feeds, Alma, GA, USA) standards and defined product specifications for protein (minimum 30%), moisture (maximum 12%), and appearance. All diets were extruded on the same day.

The amount of extruded kibble offered was initially calculated based on the metabolizable energy (ME) of the diet and the initial BW of each individual cat (1.2 × 70 (BW kg)^0.75^) and adjusted as needed based on the cat’s weekly body weight (BW) for maintenance. Proximate analysis of study diets was performed to determine crude protein, crude fat, crude fiber, moisture, and mineral analysis of calcium (Ca), phosphorus (P), manganese (Mn), sodium (Na), chloride (Cl), potassium (K), zinc (Zn), magnesium (Mg), copper (Cu), and iron (Fe) according to commercial lab standards (Table 3). Water was provided ad libitum in stainless-steel bowls to all cats throughout the study.

### 2.3. BW, BCS, Food Disappearance

Cats were fed once daily at approximately the same time and allowed 22 h to consume the allotted food. Unconsumed food was collected and weight of orts was recorded daily. Cats were weighed weekly and body condition scores (BCS; Table 4) were recorded on day 0, 45, and 90 based on the Nestlé Purina 9-point scale (1–4 = too thin; 5 = ideal; 6–9 = too heavy).

### 2.4. Anesthesia

On day 0, 45, and 90 cats were anesthetized during the sample collection procedures. Cats were administered a combination of 0.1 mL of dexmedetomidine (0.5 mg/mL) and 0.1 mL of butorphanol (10 mg/mL) intramuscularly as pre-operative sedative agents. Once the cat was sedated an intravenous injection of 4 mg/kg of 1% propofol was administered prior to intubation. Cats were intubated, administered 0.1 mL of the reversal agent atipamezole (5 mg/mL) intramuscularly, and anesthesia was maintained by inhalation of 2–3% isoflurane in oxygen. Cats were maintained under anesthesia for only the amount of time necessary to collect samples.

### 2.5. Hair Samples and Measurements

Shed hair samples were collected on day 0, 45, and 90. Prior to brushing, the brush and collection bag were weighed to determine initial weight. On day 0 cats were brushed with 10 strokes at the top of the back from the point of the shoulders to the base of the tail, approximately 15 to 29 cm in length. On day 45 and 90, cats were brushed with 20 strokes, 10 strokes at the top of the back from the point of the shoulders to the base of the tail and 10 strokes across the top of the cat along the spine from the neck to the base of the tail. Additional brush strokes were collected on days 45 and 90 compared to day 0 due to low amount of hair collected with just 10 strokes. The shed hair samples and the brush were placed in a collection bag and weighed prior to storage at room temperature.

**Figure 2:**
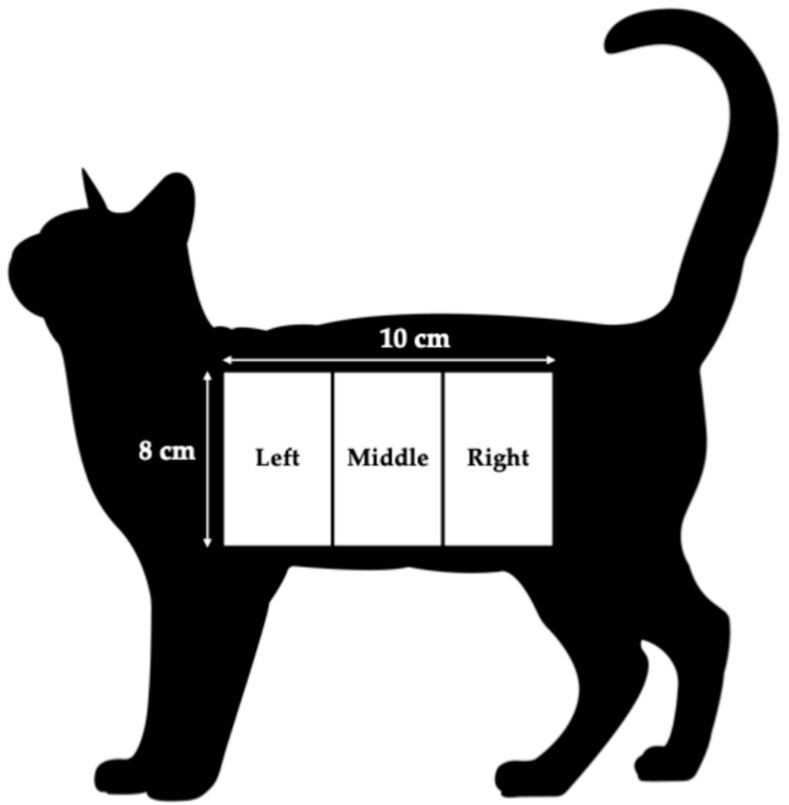
Cat hair sample and measurement diagram.

On day 0, 45, and 90, length of hair was measured to determine initial hair length and regrowth after shaving. An 8 × 10 cm area beginning from the shoulder blade and continuing 10 cm back on the side of the cat below the spine was divided into 3 regions, left, middle, and right (Figure 2). Hair length was measured from the base of the hair shaft to the tip of the hair at a 45-degree angle in the center of each region. Following initial hair length measurements on day 0, the 8 × 10 cm area was shaved with a #40 blade, allowing hair re-growth to be measured on day 45 and 90.

### 2.6. Skin and Coat Assessments

Visual coat assessments were performed on day 0, 45, and 90 by the same trained personnel for shine (0 = dull/coarse/dry; 1 = poorly reflective/non-soft; 2 = slightly reflective/somewhat soft; 3 = medium reflective/medium soft; 4 = highly reflective/very soft; 5 = greasy), dander (0 = none; 1 = fine over back; 2 = fine over body; 3 = medium over back; 4 = medium over body; 5 = severe), and overall coat quality (1 = poor; 2 = satisfactory; 3 = good; 4 = excellent).

### 2.7. Statistical Analysis

Data were analyzed with a mixed model using PROC GLIMMIX (SAS Inst., Int., Cary, NC, USA) with the cat as the experimental unit testing diet and sex; diet, sex, and day were fixed effects, as well as their interactions. Additionally, day 0 was used as a covariate for skin and coat assessments and hair measurements. Outliers were identified as observations with residuals beyond ±3 standard deviations of zero. Statistical significance was determined at *p* ≤ 0.05 and trends considered when 0.05 ≤ *p* ≤ 0.08. Means are reported as LS means.

## 3. Results

### 3.1. BW, BCS, and Food Consumption

No overall diet effects or diet × sex interactions were detected for BW, BCS, or food consumed (*p* ≥ 0.09). Male (4.5 kg) cats were heavier than female (3.7 kg) cats (*p* ≤ 0.01) throughout the trial and therefore consumed more food (*p* ≤ 0.01) as the amount of food offered was calculated based on BW to maintain consistent BCS.

### 3.2. Hair Length and Shed Hair

No overall diet effects or diet × sex interactions were detected for hair length at any time point (*p* ≥ 0.20). As expected, cats had longer hair at the end of the study period (day 90) compared to day 45 (*p* ≤ 0.01). Averaged across treatments, male cats (2.90 mm) had longer hair compared to female cats (2.59 mm) at the end of the study period (day 90, *p* ≤ 0.05).

Averaged across diet and sex, cats shed more hair at day 90 (0.102 g) compared to day 45 (0.068, *p* ≤ 0.01). No overall diet effects, sex effects, or diet × sex interactions were detected for amount of shed hair at any time point (*p* ≥ 0.44); however, a diet × day interaction was detected (*p* ≤ 0.02). Cats fed TMC-LG (0.057 g) tended to have less shed hair compared to Control-fed cats (0.080 g) at day 45 (*p* ≤ 0.08). A trend for an interaction between sex and day was identified (*p* ≤ 0.08), but post hoc analysis did not reveal any differences between males and females at day 45 or 90 (*p* ≥ 0.09) (Table 5).

### 3.3. Haircoat Evaluations

No diet, sex, or day effects, nor their interactions, were detected for overall coat quality or shine (*p* ≥ 0.25). Although no overall diet effect was detected for dander (*p* ≥ 0.57), cats fed TMC-LG had a significantly lower (*p* ≤ 0.03) dander score compared to Control-fed cats at day 45 (diet × day interaction, *p* ≤ 0.06) (Table 6).

## 4. Discussion

This study was designed to evaluate the effect of trace mineral source (inorganic vs. lysine and glutamic acid-complexed organic) on the coat quality, hair growth, and amount of hair loss (shedding) in healthy, adult shorthair cats housed indoors. The lysine and glutamic acid-complexed trace mineral source fed to the TMC-LG cats is proven to be uniquely absorbed via the amino acid transporters which renders protection from dietary antagonists such as phytic acid, folic acid, and calcium [24,25]. Cats fed TMC-LG minerals had reduced shedding and a tendency for improved dander (coat quality) scores at day 45 of the 90-day feeding trial, but the advantage dissipated by day 90.

Overall, trace mineral source did not affect BW, BCS, or food consumption which indicates that the concentration of trace minerals supplemented in this study was palatable and well-tolerated. Additionally, the difference in BW between male and female cats throughout the trial, with males being heavier, is consistent with previously published results [26,27].

There were limited effects on hair length after shaving in this study. Regardless of dietary treatment, cats had 1.35 mm longer hair at day 90 compared to day 45 and male cats had 0.31 mm longer hair than females at day 90. Baker et al. (1974) reported the average growth rate of hair for cats to be 300 microns per day with minimum growth in late winter (February—northern hemisphere) and commencement of hair growth in early summer (April/May—northern hemisphere) [27]. Similarly, Ryder (1976) and Hendriks et al. (1997) reported a sinusoidal pattern of hair growth that mirrored day length with maximum and minimum hair growth in late summer (February—southern hemisphere) and late winter (August—southern hemisphere), respectively [28,29]. Cats in these studies had access to outdoor areas and therefore were exposed to regular day length cycles throughout the year, whereas cats in the trial reported herein (December to April; northern hemisphere) were housed indoors, and the low overall amount of hair growth coincides with previous reports of minimum hair growth and follicle activity in late winter. Our data suggests that even cats housed exclusively indoors may retain innate physiological responses to day length variation throughout the year. It is also possible that the average hair growth rate estimated by Baker et al., 1974, may prove to be greater than that expected for cats housed indoors and warrants further investigation [30].

There appears to be conflicting evidence that female cats reach peak hair growth and maximum follicle inactivity earlier (~15 days) than their male counterparts [29,30]. In the current study, male cats had longer hair at the end of the trial, possibly explained by the different housing conditions and/or the large hair growth rate discrepancy between sexes.

Similarly to hair growth rate, a sinusoidal pattern of hair loss (shedding) in cats has been reported [31]. In cats with access to the outdoors, maximum hair loss was in the summer and minimum in the winter, suggesting cats are physiologically programmed to have the densest hair at the coldest time of year [31]. The last two weeks of the current study (April, Northern Hemisphere) coincide with the transition from winter to spring and thus the expected seasonal molt in cats [28]. Averaged across dietary treatments, cats in the current study had 0.034 g more hair loss at day 90 (average high temperature of 14.4 °C) compared to day 45 (average high temperature of 2.2 °C), which is consistent with a spring molt as the temperatures increased. However, at day 45 cats fed TMC-LG had 29% less hair loss compared to their Control cohorts. These data suggest that cats fed a lysine and glutamic acid-complexed organic trace mineral source had less hair loss and therefore more favorable coat density during the colder period of winter. The TM source effect on amount of shed hair did not carry through to the end of the trial as there was no significant difference in hair loss between treatments at day 90 which represented a spring transition period. A longer trial across multiple seasonal shifts to investigate the dynamic haircoat cycle of cats housed indoors is necessary to determine nutritional influence on seasonal molting patterns.

Cats in the current study were evaluated for haircoat characteristics, including shine, dander, and overall quality. Trace mineral source did not affect shine or overall coat quality. As stated previously, this study was conducted during a time of low hair follicle activity and slow hair growth; therefore, there was little opportunity to observe changes in shine or overall quality. Senior dogs fed amino acid-complexed trace minerals had significantly improved hair growth rate and coat quality, but that study was conducted in the late summer/early fall (Northern Hemisphere) [22], coinciding with seasonal patterns of hair growth and loss.

Cats fed TMC-LG had a tendency for lower dander scores compared to Control-fed cats at day 45, consistent with less hair loss at day 45. However, as with hair loss, there was no significant difference between treatments at day 90. Given the significant effect of skin integrity and overall health on coat characteristics [32], it is not surprising that TMs would play a pivotal role in haircoat maintenance. Epithelial cells are metabolically demanding and require a consistent supply of nutrients, especially trace minerals which are rate-limiting components of cellular enzymes and transcription factors regulating immunity and antioxidant status [33]. Zinc, copper, iron, and manganese are all essential for antioxidant enzymes to regulate reactive oxygen species, and when unbalanced they cause cell death and tissue damage [33,34,35]. Additionally, keratin is the primary protein in hair, nails, and the outermost layer of the epidermis. Zinc is essential for the keratinization process [15]. The results reported herein are consistent with the vital role of trace minerals in maintaining skin health, hair loss, and coat characteristics in cats. However, the explanation for the transient advantage that TMC-LG-fed cats appeared to have at day 45 in both hair loss and dander scores is not fully explained by the information collected in this study and warrants further investigation.

The authors recognize the study reported herein has some limitations. A more robust design could have included a longer test period that covered more diverse seasonal shifts; however, the study design was based on previously observed significant haircoat results in dogs fed different TM sources over a 90-day feeding trial [22]. The authors recognize that it may have been more favorable to conduct this study in the summer, a more active time of hair follicle metabolism and growth. The methodologies to measure the variables of interest in this study are qualitative but provide practical data that is directly applicable to the industry. Additionally, cats in this study were housed indoors and kept in their respective cages for the duration of the study in order to minimize aberrant hair loss. The authors recognize that this could have contributed to unintended stress that may have contributed to hair loss rates. Despite these recognized limitations, the data reported herein contributes to knowledge gaps in the literature as it relates to haircoat and hair loss responses to TM supplementation in adult, indoor-housed cats. These data also highlight the need for more research on cats housed indoors to further elucidate how haircoat cycles differ from those with access to natural light cycles and temperature fluctuations encountered outdoors.

## 5. Conclusions

The current study evaluated the effect of supplemental trace mineral source (lysine and glutamic acid-complexed versus inorganic) on hair growth, hair loss (shedding), and coat characteristics. The results reported herein revealed decreased hair loss and a tendency for improved coat dander scores at day 45 (February) of a 90-day feeding trial in cats fed TMC-LG compared to Control-fed cats, although no significant difference was detected at day 90. The reason for the transient response is not completely understood. Further investigation of trace mineral source effects on hair growth during different times of the hair cycle to determine micronutrient impact on the dynamics of haircoat characteristics and shedding in adult cats housed indoors is warranted.

## Figures and Tables

**Table 1:** Characteristics utilized to randomly assign cats to TM diet treatments ^1^.

Item	Control	TMC-LG
Animals, n	20	20
Sex, n		
Neutered males	9	9
Spayed females	11	11
Age ^2^, year	8.6	8.2

^1^ Dietary treatments were fed for a 90-day feeding trial, Control or TMC-LG formulated to iso-levels of TM (100 ppm Zn, 15 ppm Mn, 12 ppm Cu, 80 ppm Fe) as sulfates or lysine and glutamic acid-complexed TM (Zinpro Corp. Eden Prairie, MN, USA), respectively. ^2^ Age did not differ between treatments or sex, *p* > 0.1.

**Table 2:** Trace mineral inclusions for extruded kibble cat diets ^1^.

Item	Control	TMC-LG
ZnSO_4_	100 ppm	.
MnSO_4_	15 ppm	.
CuSO_4_	12 ppm	.
FeSO_4_	80 ppm	.
Zinpro^®^ ProPath^®^ Zn ^2^	.	100 ppm
Zinpro^®^ ProPath^®^ Mn ^2^	.	15 ppm
Zinpro^®^ ProPath^®^ Cu ^2^	.	12 ppm
Zinpro^®^ ProPath^®^ Fe ^2^	.	80 ppm

^1^ Dietary treatments were fed for a 90-day feeding trial, Control or TMC-LG formulated to iso-levels of TM (100 ppm Zn, 15 ppm Mn, 12 ppm Cu, 80 ppm Fe) as sulfates or lysine and glutamic acid-complexed TM (Zinpro Corp. Eden Prairie, MN, USA), respectively. ^2^ Zinpro Corporation, Eden Prairie, MN, USA.

**Table 3:** Cat extruded kibble diet composition ^1,2^.

Item	Control	TMC-LG
Dry matter, %	89.15	88.33
Crude protein, %	30.30	30.40
Fat, %	10.20	10.30
Crude fiber, %	2.30	1.15
Ash, %	8.23	8.16
NFE ^3^, %	38.12	38.32
Zinc, ppm	141.00	150.00
Manganese, ppm	36.80	37.6
Copper, ppm	19.90	20.00
Iron, ppm	183.00	186.00

^1^ Dietary treatments were fed for a 90-day feeding trial, Control or TMC-LG formulated to iso-levels of TM (100 ppm Zn, 15 ppm Mn, 12 ppm Cu, 80 ppm Fe) as sulfates or lysine and glutamic acid-complexed TM (Zinpro Corp. Eden Prairie, MN, USA), respectively. ^2^ Proximate analysis—Midwest Laboratories (Omaha, NE, USA); As-fed basis. ^3^ Calculated.

**Table 4:** The effect of TM source on body weight (BW), body condition score (BCS), and food disappearance in cats ^1,2^.

Item	Control	TMC-LG	SEM
BW, kg			
Start study period ^3^	4.1	4.1	0.1
End study period ^4^	4.1	4.2	0.1
BCS ^5^			
Start study period ^3^	5.2	5.2	0.1
End study period ^4^	5.1	5.1	0.1
Food consumption ^6^, g	83.6	80.9	2.3

^1^ Dietary treatments were fed for a 90-day feeding trial, Control or TMC-LG formulated to iso-levels of TM (100 ppm Zn, 15 ppm Mn, 12 ppm Cu, 80 ppm Fe) as sulfates or lysine and glutamic acid-complexed TM (Zinpro Corp. Eden Prairie, MN, USA), respectively. ^2^ No statistical differences between treatments. ^3^ Study day 0. ^4^ Study day 90. ^5^ Based on a 9-point scale: 1–4 = too thin; 5 = ideal; 6–9 = too heavy. ^6^ Daily average food consumed over 90-day study period.

**Table 5:** The effect of TM source on cat hair length and shed hair weight ^1^.

Item	Diet		Sex		SEM
	Control	TMC-LG	Males	Females	
Hair length ^2^, mm					
Day 45	1.45	1.34	1.42 ^c^	1.37 ^c^	0.07
Day 90	2.80	2.68	2.90 ^a^	2.59 ^b^	0.07
Shed hair ^3^, g					
Day 45 ^4^	0.080 ^b^	0.057 ^b^	0.073	0.064	0.01
Day 90	0.099 ^a^	0.104 ^a^	0.095	0.108	0.01

^1^ Dietary treatments were fed for a 90-day feeding trial, Control or TMC-LG formulated to iso-levels of TM (100 ppm Zn, 15 ppm Mn, 12 ppm Cu, 80 ppm Fe) as sulfates or lysine and glutamic acid-complexed TM (Zinpro Corp. Eden Prairie, MN), respectively. ^2^ Diet *p* = 0.197; Sex *p* = 0.045; Day *p* < 0.01; Diet × Day *p* = 0.959; Sex × Day *p* = 0.012. ^3^ Diet *p* = 0.439; Sex *p* = 0.829; Day *p* < 0.01; Diet × Day *p* = 0.025; Sex × Day *p* = 0.068. ^4^ At day 45, Control vs. TMC-LG tended to differ, *p* ≤ 0.08. ^abc^ Means within a row lacking common superscripts differ, *p* ≤ 0.05.

**Table 6:** The effect of TM source on cat coat quality ^1^, shine ^2^, and dander ^3,4^.

Item	Diet		SEM
	Control	TMC-LG	
Overall coat quality ^5^	2.75	2.78	0.08
Shine ^6^			
Day 45	2.47	2.48	0.16
Day 90	2.67	2.42	0.16
Dander ^7^			
Day 45	1.05 ^b^	0.66 ^a^	0.12
Day 90	0.80 ^b^	0.86 ^b^	0.12

^1^ 0 = dull/coarse/dry; 1 = poorly reflective/non-soft; 2 = slightly reflective/somewhat soft; 3 = medium reflective/medium soft; 4 = highly reflective/very soft; 5 = greasy. ^2^ 0 = none; 1 = fine over back; 2 = fine over body; 3 = medium over back; 4 = medium over body; 5 = severe. ^3^ 1 = poor; 2 = satisfactory; 3 = good; 4 = excellent. ^4^ Dietary treatments were fed for a 90-day feeding trial, Control or TMC-LG formulated to iso-levels of TM (100 ppm Zn, 15 ppm Mn, 12 ppm Cu, 80 ppm Fe) as sulfates or lysine and glutamic acid-complexed TM (Zinpro Corp. Eden Prairie, MN, USA), respectively. ^5^ Diet *p* = 0.786; Sex *p* = 0.947; Day *p* = 0.629; Diet × Day *p* = 0.253. ^6^ Diet *p* = 0.567; Sex *p* = 0.748; Day *p* = 0.270; Diet × Day *p* = 0.056. ^7^ Diet *p* = 0.302; Sex *p* = 0.386; Day *p* = 0.776; Diet × Day *p* = 0.007. ^ab^ Means lacking common superscripts differ, *p* ≤ 0.05.

## Data Availability

Restrictions apply to the availability of these data. Data were obtained by the study sponsor and may be available at the discretion of the study sponsor.

## Abbreviations

The following abbreviations are used in this manuscript:

TM	Trace mineral
Zn	Zinc
Mn	Manganese
Cu	Copper
Fe	Iron
AAFCO	Association of American Feed Control Officials
BCS	Body condition score
BW	Body weight
ME	Metabolizable energy
